# Redox Systems Biology in Chronic Kidney Disease

**DOI:** 10.1155/2023/9864037

**Published:** 2023-05-03

**Authors:** Stefanos Roumeliotis, Vassilios Liakopoulos, Andrej Veljkovic, Evangelia Dounousi

**Affiliations:** ^1^Division of Nephrology and Hypertension, 1st Department of Internal Medicine, AHEPA Hospital, School of Medicine, Aristotle University of Thessaloniki, 54636 Thessaloniki, Greece; ^2^Faculty of Medicine, University of Nis, 18000 Nis, Serbia; ^3^Department of Nephrology, School of Medicine, University of Ioannina, Ioannina, Greece

Chronic kidney disease (CKD) is a gradual, progressive disease affecting more than 11% of the general population and approximately 844 million subjects globally [1]. CKD has emerged as a significant noncommunicable risk for mortality with increasing prevalence and incidence; since 1990, the all-age death rate caused by CKD was increased by 42% worldwide [2]. Therefore, it became evident that it is of utmost importance to fully elucidate the exact mechanisms driving the onset and progression of CKD. Among these, oxidative stress (OS), resulting from the imbalance between prooxidant and antioxidant molecules, is a novel pathophysiological pathway underlying the progression of CKD and cardiovascular disease (CVD).

Although OS and inflammation have been long identified as risk factors for the onset and progression of CVD in patients with renal impairment, biomarkers of these conditions have not yet been utilized in everyday clinical practice. To investigate the possible predictive value of inflammation and OS on cardiac necrosis and function, S. Roumeliotis et al. enrolled 100 patients presented with NSTEMI (non-ST-elevation myocardial infarction) and impaired kidney function in the emergency care unit and found that circulating MPO (myeloperoxidase), IL-10 (interleukin-10), and high-sensitive CRP (C-reactive protein) identified patients with low ejection fraction and high troponin (area under the curve 0.67, receiver operator characteristic (ROC) analysis) [3]. Moreover, cardiac function deteriorated and inflammation markers were increased progressively with CKD progression. The authors suggested that markers of OS and inflammation could be useful for predicting clinical outcomes in these patients.

Diabetic kidney disease (DKD) is a highly heterogenous disease, including both the traditional proteinuric and the novel, nonproteinuric phenotype. Since type 2 diabetes mellitus still remains the leading cause of ESKD worldwide and the annual incidence rates of DKD-derived ESKD continue to rise, it is crucial to identify the biological pathways that drive the progression of the disease. The pathophysiology of DKD is rather complex and includes several molecular pathways such as autophagy, cell hypoxia, activation of protein kinase C, polyol and hexosamine, overproduction of advanced glycation end-products (AGEs), alterations in kidney hemodynamics, systemic hypertension and mitochondria, urinary micro-RNAs, and sodium-glucose cotransporter-2 [4]. Among these pathways, OS is implicated in the pathogenesis and progression of DKD. Until to date, the studies investigating the possible role of susceptibility genes in the pathway of OS-derived DKD remain limited and have produced controversial results. To explore this area, A. Roumeliotis et al. conducted a case-control association study and enrolled 341 diabetes mellitus type 2 (DM2) divided into two groups: 121 presenting with DKD and 220 with normal kidney function, as defined by normal estimated glomerular filtration rate and no albuminuria, which served as controls [5]. In this population, genome-wide association analysis revealed 43 single nucleotide polymorphisms (SNPs) involved in the OS pathway playing either a protective or a contributing role in the onset of DKD. The corresponding encoding genes of these SNPs were SPP1, TPO, TTN, SGO2, NOS3, PDLIM1, CLU, CCS, GPX4, TXNRD2, EPHX2, MTL5, EPX, GPX3, ALOX12, IPCEF1, GSTA, OXR1, GPX6, AOX1, and PRNP gene. The authors suggested that there might be a genetic background of OS in the pathophysiology of DKD in DM2 subjects. In agreement with these findings, C. Y. Kuo et al. showed that mutations in the gene von Hippel-Lindau trigger the overproduction of inflammatory cytokines and ROS in kidney tubular cells and might predispose to the development of renal cell carcinoma [6], thus highlighting the clinical importance of genetic background.

The pivotal role of OS in DKD was also highlighted by another prospective study with long follow-up by the same group of authors [7]. In a cohort of 91 proteinuric DKD patients, followed for a period of 10 years, high-oxidized low-density lipoprotein (ox-LDL) at baseline was associated with the primary endpoint of death or at least a 30% decrease in eGFR, or progression to ESKD (*p* = 0 : 001, log-rank test and HR = 3 : 42, 95% CI = 1 : 55–7 : 56, *p* = 0 : 002, Cox regression analysis). Furthermore, high baseline ox-LDL was a better predictor of eGFR decline over this period of time compared to proteinuria (AUC 71%, 95% CI = 0 : 59–0 : 83, *p* = 0 : 001, versus AUC 67%, 95% CI = 0 : 54–0 : 79, *p* = 0 : 014, respectively). Taken together, these two studies suggest that OS plays a pivotal role in both the onset and progression of DKD and could be regarded as a novel risk factor in these patients.

At a molecular level, the mitochondria-associated endoplasmic reticulum (ER) membrane (MAM) might play a crucial role in DKD. MAM participates in several cellular activities including calcium signaling [8], biosynthesis and transfer of lipids [9], ER stress response [10], autophagy, and dynamic alterations in mitochondria [11]. ER might become severely stressed due to hyperglycemia, accumulation of ROS and AGEs, fatty acids, and activation of the angiotensin II receptor pathway [12]. Through these functions, MAM is involved in the regulation of lipid accumulation, calcium overload, and kidney tubule cell apoptosis, thus contributing to OS and inflammation in DKD [13]. To combat the deleterious effects of OS endogenous- or exogenous-administered antioxidant molecules attack to ROS, neutralize them and thus might ameliorate the clinical OS-derived adverse clinical outcomes, including CV events [14] and progression of DKD [15, 16]. One of the novel proposed mechanisms driving CKD progression is mitochondrial dysfunction. It is believed that the injury of mitochondria might trigger the overproduction of ROS and therefore podocyte apoptosis [17, 18], and experimental data suggest that mitoquinone (MitoQ), a highly selective antioxidant targeting mitochondria, might protect angiotensin II-derived mitochondrial damage in podocytes through the Keap1 (Kelch-like ECH-associated protein 1) Nrf2 (nuclear factor E2-related factor 2) signaling pathway [19].

Besides OS, inflammation also plays a pivotal role in the pathogenesis of CKD. OS triggered by methylglyoxal (MGO), a highly reactive carbonyl, increases local inflammation in kidney cells. *Cudrania tricuspidata* (CT) is a natural endogenous antioxidant, containing flavonoids, phenolic compounds, and xanthones. In East Asia, it has been long used as a traditional remedy for inflammation and tumors [20, 21]. D. Kim et al. performed an experimental study to investigate whether a CT root extract (CTRE) might abrogate MGO-induced ROS production and inflammation in kidney cells [22] and found that this antioxidant suppressed MGO-derived OS and inflammation through the overexpression of the Nrf2 antioxidant agent and downregulation of NADPH oxidase 4 (NOX4) and protein kinase C (PKC) activation. However, to draw a more definite conclusion regarding the possible antioxidative and anti-inflammatory properties, this agent should be assessed in human studies.

In this special issue, we aimed to shed some light on the complex pathophysiology of OS and determine the effects of redox biology systems, OS, inflammation, and endothelial dysfunction in uremia. Until to date, the majority of evidence is obtained by experimental data or small, observational studies. To fully comprehend the clinical importance of OS and draw more definite conclusions regarding the potential beneficial effects of various antioxidants, future, large, well-designed, prospective human studies are needed.

*Stefanos Roumeliotis* *Vassilios Liakopoulos* *Andrej Veljkovic* *Evangelia Dounousi*
